# The Role of Microbes in Surgical Decision Making for Infective Endocarditis: Mitral Valve Repair or Replacement?

**DOI:** 10.3390/microorganisms12071320

**Published:** 2024-06-28

**Authors:** Elisa Mikus, Mariafrancesca Fiorentino, Diego Sangiorgi, Renato Pascale, Antonino Costantino, Chiara Nocera, Simone Calvi, Elena Tenti, Elena Tremoli, Alberto Tripodi, Carlo Savini

**Affiliations:** 1 Cardiovascular Department, Maria Cecilia Hospital, GVM Care & Research, 48033 Cotignola, Ravenna, Italy; francescafiorentino@hotmail.it (M.F.); dsangiorgi@gvmnet.it (D.S.); antonino@costantinorc.com (A.C.); scalvi@gvmnet.it (S.C.); etenti@gvmnet.it (E.T.); etremoli@gvmnet.it (E.T.); albertotripodi@hotmail.com (A.T.); carlo.savini@unibo.it (C.S.); 2 Department of Medical and Surgical Sciences, Alma Mater Studiorum University of Bologna, 40138 Bologna, Italy; renato.pascale2@unibo.it; 3 Infectious Diseases Unit, Department for Integrated Infectious Risk Management, IRCCS Azienda Ospedaliero-Universitaria di Bologna, 40138 Bologna, Italy; 4 Cardiac Surgery Department, IRCCS Azienda Ospedaliera-Universitaria di Bologna, 40138 Bologna, Italy; chiara.nocera@outlook.it; 5 Department of Experimental Diagnostic and Surgical Medicine (DIMEC), University of Bologna, 40126 Bologna, Italy

**Keywords:** bacterial endocarditis, mitral valve, cardiac surgical procedures, mitral valve repair, mitral valve replacement

## Abstract

Background: The benefits of mitral repair versus replacement for endocarditis are inconclusive. This study compares outcomes of patients with infective endocarditis undergoing mitral valve repair versus replacement and investigates the impact of microbial etiology. Methods: All 251 patients undergoing mitral valve surgery for active endocarditis between 2010 and 2023 were enrolled, 180 (71.7%) replacement and 71 (28.3%) repair. To adjust for imbalances, inverse probability of treatment weighting was applied and 187 patients were obtained. Results: The analysis between groups, following the application of inverse probability of treatment weighting, showed no statistically significant differences across all considered outcomes. Early and late death was observed respectively in 6 (8.5%) and 11 (15.5%) patients in the repair group versus 24 (13.3%) and 45 (25.0%) in the replacement group without statistical significance (*p* = 0.221 and *p* = 0.446). Relapse occurred in six patients (8.5%) in the repair group after a median time of 4.0 months and in six (3.3%) in the replacement after 6.9 months (*p* = 0.071). Conclusions: Surgical strategy in mitral endocarditis has no effect on major postoperative complications, mortality, or medium/long-term survival. *Staphylococcus aureus* and *Coagulase-negative Staphylococci* represent a risk for early mortality and relapse. However, mitral valve repair for endocarditis can be pursued when it ensures the complete eradication of all infected tissue, particularly in cases caused by *Streptococcus* infection, in young patients, and after a minimum of 18 days of antibiotic therapy.

## 1. Introduction

Infective endocarditis (IE) is a life-threatening condition characterized by the infection of the heart valves and endocardium, leading to significant morbidity and mortality [1]. This condition primarily affects the mitral valve, followed by the aortic, tricuspid, and pulmonary valves, with the potential involvement of supporting structures. In non-infective scenarios, mitral valve repair (MVr) is generally considered superior to mitral valve replacement (MVR) due to better early and long-term outcomes. Surgical intervention for IE aims to excise infected structures and restore anatomical and hemodynamic functionality. The decision between repair and replacement is influenced by the extent of damage, the acuity of the disease, and patient-specific factors such as the presence of indwelling devices, a history of drug abuse, or periodontal disease [2,3].

The recent guidelines from the European Society of Cardiology (ESC) [4] and the American Association for Thoracic Surgery [5] recommend mitral valve repair when durable results are anticipated and complete eradication of infected tissue is feasible. These guidelines highlight the importance of preserving native valve structures whenever possible. However, the comparative benefits of mitral repair versus replacement in the context of endocarditis remain inconclusive, particularly regarding recurrence rates, freedom from reoperation, and long-term survival.

A notable registry study on mitral repair versus replacement in IE, conducted by Nana Toyoda et al. in 2017 [6], indicated that mitral valve repair is associated with superior survival rates and lower risks of recurrent infection compared to valve replacement. This study advocates for the preferential use of mitral valve repair when feasible, emphasizing its potential benefits in improving patient outcomes. However, it is important to acknowledge the limitations of this study, including missing data on the severity of IE and disparities between the study groups, which may affect the consistency and generalizability of these findings.

The current study aims to elucidate the relationship between pathogens, preoperative echocardiographic findings, and surgical interventions in patients undergoing mitral valve repair. By examining these factors, this study seeks to provide a comprehensive understanding of the impact of different pathogens and preoperative conditions on surgical outcomes. Additionally, this study will assess the incidence of mortality and relapse (both early and late) in this population, providing valuable insights into the long-term outcomes of mitral valve repair in patients with infective endocarditis. This information could inform clinical decision-making and contribute to the development of more effective treatment strategies for this serious condition.

## 2. Materials and Methods

Demographic and major baseline characteristics of the patients were collected and stored. These characteristics included age, sex, body mass index, creatinine clearance, preoperative condition, cardiovascular risk factors, functional status, and left ventricular ejection fraction, as well as the EuroSCORE II, which provides an assessment of the risk of mortality from cardiac surgery. In addition to these baseline characteristics, data on intraoperative and short-term outcomes were also compiled.

To evaluate cardiac function and the effectiveness of the surgical interventions, echocardiography was performed both preoperatively and postoperatively. This imaging technique provided crucial insights into the structure and function of the heart valves, as well as overall cardiac performance before and after surgery.

Clinical follow-up of the patients was conducted at two key intervals: 30 days post-surgery and after a mean follow-up period of 10.3 months. These follow-up evaluations were carried out through telephone interviews or in-person patient visits, ensuring comprehensive monitoring of the patients’ recovery and long-term health outcomes.

### 2.1. Study Design and Outcomes

Between January 2010 and December 2023, a total of 14,532 patients underwent surgical cardiac operations at our center. Out of these, 3823 patients received treatments involving mitral valve replacement or repair. This monocentric, retrospective study specifically focuses on 251 patients who underwent either mitral valve replacement or repair due to endocarditis. A detailed flowchart of the included patients is available in the Supplementary Materials (Figure S1). No formal sample size calculation was performed; instead, all eligible patients within the specified timeframe were included in the study.

The study protocol received approval from the Romagna Ethics Committee on 30 June 2023 (protocol number 4497/2023 I.5/95). Given the retrospective nature of the data collected, individual informed consent was waived. Data collection commenced from clinical charts and was systematically collected in a specific registry. Extensive efforts were made to minimize the occurrence of missing information. In instances where data were missing, it was due to the nature of gaps in clinical documentation and assumed to be missing completely at random. Consequently, only complete cases were included in the analysis to maintain data integrity.

The patient cohort was divided into two groups based on the surgical technique employed. The first group consisted of 180 patients (71.7%) who underwent mitral valve repair. The second group comprised 71 patients (28.3%) who underwent mitral valve replacement. Detailed demographic and clinical characteristics of these groups are summarized in Table 1.

### 2.2. Statistical Analysis

After assessing normality with the Shapiro–Wilk test, continuous variables were presented as the median and interquartile range (IQR) and compared across groups using the Mann–Whitney test. Categorical variables were presented as absolute numbers and frequencies and compared using the Chi-squared test or Fisher’s exact test, as appropriate. To mitigate selection bias and ensure a more accurate comparison between groups, inverse probability of treatment weighting (IPTW) was employed. Baseline and intra-operative characteristics were utilized to construct the weights for all weighted analyses. Observations that fell outside the common support area, where the propensity scores of treated and untreated subjects do not overlap, were excluded from the analysis to avoid bias from extreme values. Absolute standardized mean differences (ASMD) were reported to evaluate balance across groups, with variables having an ASMD < 0.2 considered balanced [7]. The weighted means and percentages after IPTW were also calculated. Weighted Kaplan Meier curves for death during follow-up were reported and compared with the weighted log-rank test. The probability of relapse was assessed using the cumulative hazard function with the Fine–Gray method, using death as a competing risk event. A propensity-adjusted Cox model was also performed in order to assess the influence of each pathogen on death.

Weighted logistic regression models and Generalized Linear Models (GLMs) with gamma or negative binomial distribution and log link function were used, respectively, to assess differences in binary or continuous in-hospital complications; deviance residuals were analyzed for normality.

Multivariable logistic regression was also assessed in order to identify factors that led to the MVr decision; Akaike Information Criteria (AIC) minimization was used for model selection; the area under the ROC curve was reported in order to assess model discrimination; Youden’s index was used to identify an optimal cut point for the distance in days between endocarditis onset and the moment of surgery. To reduce missing bias, multiple imputation by predictive mean matching (PMM) with 10 replications was used to impute missing values on the only one covariate with missing values (distance endocarditis-surgery, which presented 21 missing values, 8.4%). All analyses were performed with STATA 18.0 SE (StataCorp LLC, College Station, TX, USA); *p*-values < 0.05 were considered statistically significant.

## 3. Results

The baseline characteristics of the whole patient population, divided in two groups according to surgical treatment, are reported in Table 1 and Table S1 (Supplementary Materials).

**Figure 1:**
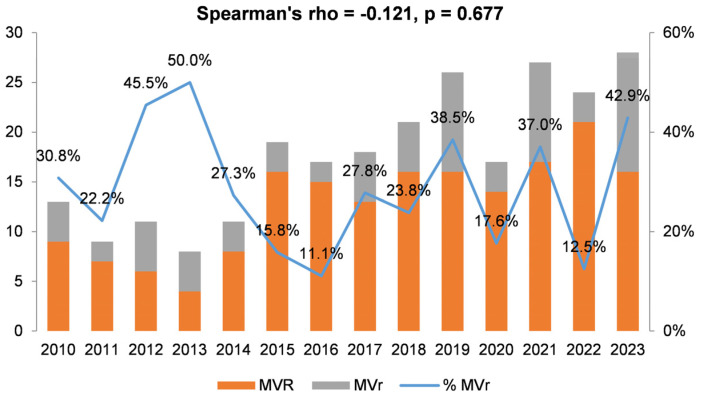
Time series of surgical procedures mitral valve repair or replacement.

A total of 251 patients with either active or healed mitral valve endocarditis were included in our study: 180 (71.7%) underwent mitral valve replacement, and the remaining 71 (28.3%) underwent mitral valve repair. The repair rate for mitral valve endocarditis has remained stable over time (*p* = 0.677), as illustrated in Figure 1. Patients with multi-valve disease were also included.

There were significant differences between the two groups. In detail, patients who received a mitral valve repair were younger (*p* = 0.013), with a lower EuroSCORE additive and logistic (respectively, *p* = 0.001 and 0.004 and without abscess (*p* < 0.001). In this population of patients undergoing mitral valve repair, *Streptococcus* was the most frequent pathogen (31.3%; *p* = 0.050, borderline non-significant), in contrast to *Staphylococcus aureus*, which was the most frequent pathogen in patients who underwent mitral valve replacement. In addition, patients treated with mitral repair underwent surgical operation after a longer period of time from the diagnosis and the beginning of antibiotic therapy, with a statistically significant difference in the interval of time between diagnosis and surgery (*p* = 0.010) (Table 1).

In order to reduce selection bias, inverse probability of treatment weighting (IPTW) calculated from the propensity score (PS) was applied and a population of 187 patients was obtained (Table 1).

Table 2 describes the echocardiographic findings in two groups, which were also included in IPTW.

### 3.1. Microbial Etiology

**Figure 2:**
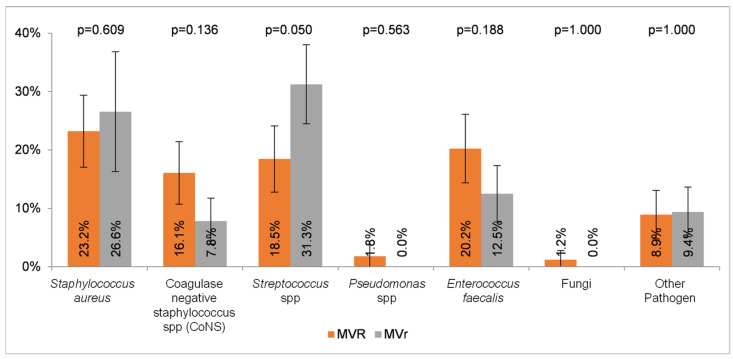
Gram-positive etiology distribution in patients undergoing MVR or MVr.

Regarding microbial etiology, *Staphylococcus aureus* was the main pathogen responsible for IE in our cohort (56, 22.3%), followed by *Streptococcus* spp. (51, 20.3%), *Enterococcus faecalis* (42, 16.7%), and coagulase-negative staphylococci spp. (CoNS), n (32, 12.7%). No differences were found among Gram-positive etiology distribution in patients undergoing MVR or MVr (Figure 2).

**Figure 3:**
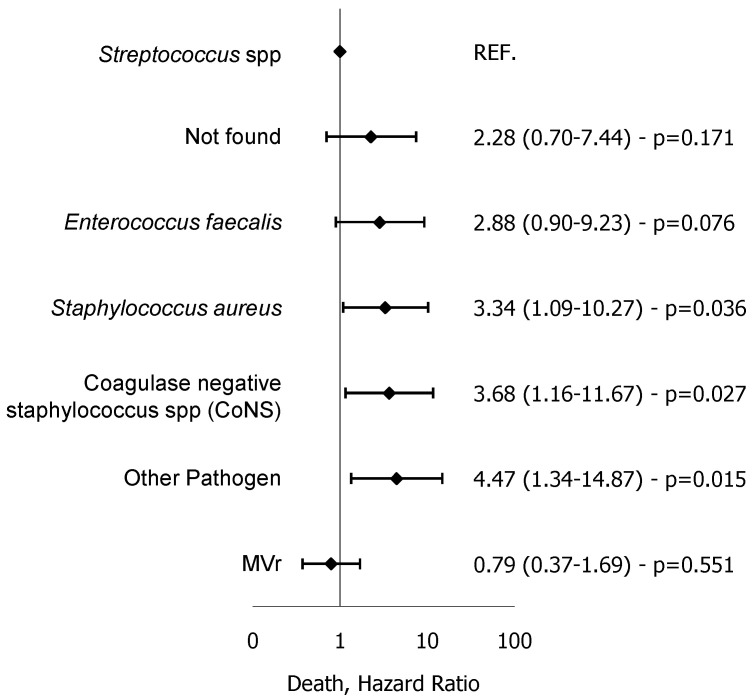
Forest plot for multivariable proportional hazards Cox regression model for all-cause mortality.

A limited number of patients had IE due to *Pseudomonas* spp. and Fungi, 3 (1.2%) and 2 (0.8%), respectively, and all were in the MVR group. At multivariable analysis for all-cause mortality, *Staphylococcus aureus* (HR = 3.34), CoNS (HR = 3.68) and other pathogens (including *Pseudomonas* spp. and fungi, HR = 4.47) were independent risk factors.(Figure 3).

### 3.2. Postoperative Outcomes and Mortality

The surgical technique adopted for mitral valve repair or replacement included both traditional median sternotomy (n = 210) or minimally invasive right minithoracotomy (n = 41).

Surgical data, which were also incorporated in IPTW, are reported in Table 3.

All postoperative outcomes are described in Table 4 and Table S2 (Supplementary Materials). Before IPTW, the worst outcome for mitral valve replacement was observed in terms of ventilation time, ICU, complications, low cardiac output, and respiratory failure. The analysis between groups after the inverse probability of treatment weighting (IPTW) showed no statistically significant differences in all considered outcomes.

Early death (in-hospital or within 30 days mortality) was observed in 6 patients (8.5%) in the repair group and 24 (13.3%) in the replacement group. After the inverse probability of treatment weighting, no differences were observed (*p* = 0.221).

### 3.3. Multivariable Logistic Regression Analysis

Using multivariable logistic regression analysis, we found that factors supporting a mitral valve repair, instead of a replacement, are the infection caused by *Streptococcus* (OR = 2.261), the age (OR = 0.978) (for each additional year of age, the probability of mitral valve repair decreases by 2%), and the dichotomized interval of time between diagnosis and surgery (OR = 2.916) (area under curve 69.4%). Youden’s index identified an optimal cut-point for interval of time between diagnosis and surgery ≥ 18 days in relation to valve repair; the area under the ROC curve was 60.8% (Table 5 and Figure S2, Supplementary Materials).

### 3.4. Recurrence of Infective Endocarditis and Late Mortality

**Figure 4:**
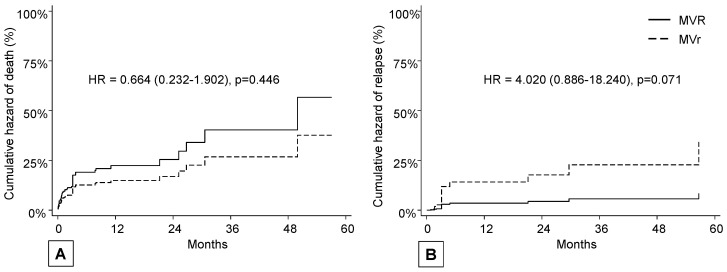
(**A**) cumulative hazard of death; (**B**) cumulative hazard of recurrence of infective endocarditis (death as a competing risk event).

Death occurred in 11 patients (15.5%) in the repair group after a median time of 0.8 months and in 45 (25.0%) in the replacement group after a median follow-up of 3.1 months (mean follow-up of 10.3 months), with no differences in terms of hazard ratio (*p* = 0.446) (Figure 4, panel A).

Relapse occurred in six patients (8.5%) in the repair group after a median time of 4.0 months and in six (3.3%) in the replacement group after a median follow-up of 6.9 months; even if not statistically significant, a trend was observed in favor of MVR in terms of hazard ratio (*p* = 0.071) (Figure 4, panel B).

## 4. Discussion

In this study, we compared the survival rates of patients who underwent mitral valve repair with that of patients treated with replacement for endocarditis. Historically, both American and 2017 European consensus guidelines recommend mitral valve repair over replacement for the surgical management of active mitral native valve endocarditis, based on the results from single-center case series [5,8,9]. However, new European guidelines published in 2023 cast doubt on this recommendation, emphasizing the importance of eradicating all infected tissue rather than focusing solely on repair [4]. Consequently, mitral valve replacement remains the most common surgical intervention for mitral valve infective endocarditis, with approximately 20–30% of cases of repair, as reported by national registries and consistent with our findings [6,10,11,12].

We observed significant disparities between the two groups. Indeed, patients in the mitral replacement cohort tended to be older, sicker, and presented with more severe lesions/abscesses and heart failure compared with those in the repair cohort. Consequently, patients in the replacement group experienced a higher rate of complications.

To mitigate selection bias, we employed inverse probability of treatment weighting based on propensity scores, including echocardiographic findings, to create a more homogeneous study population. In this subgroup, outcomes in terms of both in-hospital mortality and complications were comparable. However, younger patients infected with *Streptococcus* spp. and with antibiotic therapy initiated at least 18 days prior to surgery tended to fare better with repair, provided their clinical condition allowed for it.

Patients with mitral valve endocarditis are a highly heterogeneous group both clinically and echocardiographically, making it challenging to formulate definitive treatment recommendations. As regards the etiology, we confirmed a known epidemiology of infective endocarditis in our cohort. *Staphilococcus aureus* and CoNS were the most frequently isolated organisms in patients belonging to high-risk groups such as those on hemodialysis, intravenous drug users, and patients with permanent lines and devices. Conversely, streptococcal infective endocarditis has been diagnosed in patients with oral, gastrointestinal, and urogenital tract changes, but often usually younger and in relatively good health. This aspect must be considered in the indication to establish the urgency of the surgical intervention and the consequent prolongation of the preoperative antibiotic course [13].

Additionally, the feasibility of valve repair in infected individuals varies due to technical constraints. While many authors argue for the superiority of repair over replacement in terms of both early and late outcomes, the heterogeneity of the patient population, the extent of infected lesions, and causative pathogens prevent the generalization of these findings. Absolute superiority of repair over replacement in terms of long-term survival has yet to be demonstrated and lacks conclusive evidence [11,14,15]. In the whole population, no relapse case was observed in patients affected by *Streptococcus* spp. endocarditis. Moreover, in the mitral valve repair population, 25% of endocarditis with unknown microbial etiology relapsed and this might be explained by the lack of targeted antimicrobial therapy. Nevertheless, considering the low number of relapse events, it is not possible to draw conclusions on the best surgical treatment to be adopted. In the future, it could be interesting to investigate the role of proactive action in reducing the bacterial load and modulating the microbiome of the oral cavity (such as oral hygiene using probiotics) and the consequent impact on the required surgical technique [16,17].

The in-hospital mortality of infective endocarditis is estimated at around 20%, although this figure varies substantially according to the infecting organism. *Staphylococcus aureus* is one of the most important and well-known adverse prognostic factors for patients with IE [13]. However, similar rates of mortality in patients with CoNS and patients with *Staphylococcus aureus* IE have been reported. In a study on native valve IE, Chu et al. showed a significantly higher mortality rate of patients with *Staphylococcus aureus* and CoNS etiology compared with patients with *Streptococcus* spp. [18]. Similarly, fungal and Gram-negative endocarditis, even if relatively rare, are both associated with high morbidity and mortality [19,20]. Patients with IE due to *Staphylococcus aureus*, CoNS, *Pseudomonas* spp., and fungi compared to patients with IE due to *Streptococcus* spp. However, we did not find any association between microbial etiology and recurrence of infection according to the type of surgery performed, probably due to the relatively limited sample size.

### Limitations

This study has several limitations. Firstly, it is non-randomized, and variables were collected retrospectively. The decision between mitral repair and replacement depended on various factors and was at the discretion of the surgeon. There are differences between the populations undergoing mitral replacement and mitral repair in terms of preoperative clinical status and disease extent. In addition, this study reflects the experience of a single surgical center without a formal sample size calculation.

## 5. Conclusions

To date, mitral valve repair is widely recognized as the preferred treatment for degenerative disease, but uncertainty remains regarding its efficacy in cases of infective endocarditis. The data reported in this study show that the choice of surgical strategy for infective endocarditis does not significantly impact major postoperative complications, mortality, or medium/long-term survival. In addition, mitral valve repair shows a trend toward a higher incidence of relapse, probably related to the high percentage of cases with unknown microbial etiology and consequently without targeted antimicrobial therapy. Of importance is the observation that both *Staphylococcus aureus* and coagulase-negative staphylococci represent a risk for early mortality and relapse. However, mitral valve repair for endocarditis can be pursued when it ensures the complete eradication of all infected tissue, particularly in cases caused by *Streptococcus* infection, in young patients, and after a minimum of 18 days of antibiotic therapy.

## Figures and Tables

**Table 1:** Preoperative characteristics.

	MVR	MVr	*p*	MVR, Weighted %/Mean	MVr, Weighted %/Mean	ASMD Post IPTW
N	180	71		120	67	120 vs. 67
Age, median (IQR)	69 (62–76)	62 (54–73)	0.013	63.1	60.4	0.179
Female, n (%)	78 (43.3)	26 (36.6)	0.394	39.0	45.5	0.134
Endocarditis Site, n (%)			0.237			0.022
-Native Valve	112 (62.2)	50 (70.4)		68.3	67.0	
-Native Valve + Prosthesis	14 (7.8)	7 (9.9)		8.7	9.6	
-Prosthesis	54 (30.0)	14 (19.7)		23.0	23.5	
Negative blood culture or sample culture, n (%)	18 (10.0)	9 (12.7)	0.508	11.3	13.2	0.059
*Staphylococcus aureus*, n (%)	39 (23.2)	17 (26.6)	0.609	18	21.1	0.077
Coagulase negative staphylococcus spp. (CoNS), n (%)	27 (16.1)	5 (7.8)	0.136	9.4	11.5	0.068
*Streptococcus* spp., n (%)	31 (18.5)	20 (31.3)	0.050	22.4	18.6	0.093
*Pseudomonas* spp., n (%)	3 (1.8)	0 (0.0)	0.563	2.2	0.0	0.017
*Enteroccoccus Feacalis*, n (%)	34 (20.2)	8 (12.5)	0.188	15.1	12.1	0.088
Fungi, n (%)	2 (1.2)	0 (0.0)	1.000	1.4	0.0	0.017
Other Pathogen, n (%)	15 (8.9)	6 (9.4)	1.000	10.7	11.2	0.017
Drug addiction, n (%)	6 (3.3)	7 (9.9)	0.054	7.8	5.6	0.088
Additive Euroscore, median (IQR)	10 (8–13)	8 (5–11)	0.001	/	/	/
Logistics Euroscore, median (IQR)	20.0 (10.0–35.2)	11.5 (4.4–32.2)	0.004	19.7	19.8	0.005

IQR, interquartile range; MVR, mitral valve prosthesis; MVr, mitral valve repair.

**Table 2:** Echocardiographic findings.

	MVR	MVr	*p*	MVR, Weighted %/Mean	MVr, Weighted %/Mean	ASMD Post IPTW
N	180	71		120	67	120 vs. 67
Abscess, n (%)	35 (19.4)	2 (2.8)	<0.001	5.9	11.1	0.155
Vegetations, n (%)	151 (83.9)	56 (78.9)	0.360	82.5	83.8	0.033
Flap perforation, n (%)	40 (22.2)	19 (26.8)	0.509	23.3	18.9	0.102
Prosthesis Detachment, n (%)	34 (18.9)	9 (12.7)	0.270	14.6	14.2	0.010
Distance Endocarditis-Surgery, Days, median (IQR)	21 (13–36)	29 (18–50)	0.010	33.1	30.5	0.065

**Table 3:** Surgical data.

	MVR	MVr	*p*	MVR, Weighted %/Mean	MVr, Weighted %/Mean	ASMD Post IPTW
N	180	71		120	67	120 vs. 67
Full Sternotomy, n (%)	158 (87.8)	52 (73.2)	0.008	81.5	79.6	0.048
N. Treated Valves, median (IQR)	1 (1–2)	2 (1–2)	0.019	1.5	1.5	0.134
Concomitant CABG, n (%)	16 (8.9)	2 (2.8)	0.902	4.1	4.5	0.016
Ascending Aorta replacement, n (%)	4 (2.2)	5 (7.0)	0.123	/	/	/
CPB time, median (IQR)	113 (85–141)	118 (93–151)	0.224	119	118	0.025
Cross Clamp Time, median (IQR)	93 (68–115)	97 (77–130)	0.196	/	/	/
Concomitant AVR, n (%)	64 (35.6)	39 (54.9)	0.007	48.0	42.1	0.119
Antibiotic therapy (>6 week) n (%)	36 (20.0)	21 (29.6)	0.132	19.1	18.9	0.005

AVR, aortic valve replacement, CABG, coronary artery bypass grafting; CPB, cardiopulmonary bypass; IQR, interquartile range; MVR, mitral valve prosthesis; MVr, mitral valve repair.

**Table 4:** Postoperative outcomes.

	MVR	MVr	Coef. * (95%CI), *p*
Ventilation time (hours), median (IQR)	12 (6–45)	10 (6–14)	−0.033 (−0.520; 0.455), *p* = 0.896
ICU (Days), median (IQR)	4 (2–7)	2 (2–5)	−0.001 (−0.375; 0.374), *p* = 0.997
Complications, n (%)	111 (61.7)	32 (45.1)	0.770 (0.359; 1.652), *p* = 0.502
Sepsis, n (%)	17 (9.4)	7 (9.9)	2.215 (0.568; 8.630), *p* = 0.252
Hospital stay (days), median (IQR)	8 (7–12)	8 (6–11)	0.081 (−0.228; 0.390), *p* = 0.608
Early death, n (%)	24 (13.3%)	6 (8.5%)	0.485 (0.152–1.545), *p* = 0.221

ICU, intensive care unit; IQR, interquartile range; MVR, mitral valve prosthesis; MVr, mitral valve repair. * Model coefficient is β for continuous outcomes (ventilation time, ICU days, hospital stay) and derived from weighted GLM gamma or negative binomial models; all others are odds ratios derived from weighted logistic regression models.

**Table 5:** Factors supporting a mitral valve repair.

	OR	95% CI		*p*
Endocarditis-surgery, ≥18 days	2.916	1.488	5.713	0.002
Age	0.978	0.958	0.999	0.040
*Streptococcus* spp.	2.261	1.134	4.510	0.021
Chronic obstructive pulmonary disease	0.445	0.159	1.249	0.124
Drug addicted	2.783	0.812	9.544	0.103

## Data Availability

The data presented in this study are available on request from the corresponding author. The data are not publicly available due to Data Protection Directive 95/46/EC.

## Supplementary Materials

The following supporting information can be downloaded at: https://www.mdpi.com/article/10.3390/microorganisms12071320/s1, Figure S1. Consortium flowchart of included patients, Figure S2. Receiver operator characteristic for the time between diagnosis and surgery in relation to valve repair/replacement, Table S1: Baseline demographic and clinical characteristics. Table S2. Postoperative outcomes.
