# Supplementary material for: The Role of Microbes in Surgical Decision Making for Infective Endocarditis: Mitral Valve Repair or Replacement?
Source: Microorganisms. 2024 Jun 28;12(7):1320. doi: 10.3390/microorganisms12071320 (PMC11278633; doi:10.3390/microorganisms12071320)
Supplement: Supplementary file 1 [file microorganisms-12-01320-s001.zip › microorganisms-3061537-supplementary.pdf]

Figure S1. Consortium flowchart of included patients

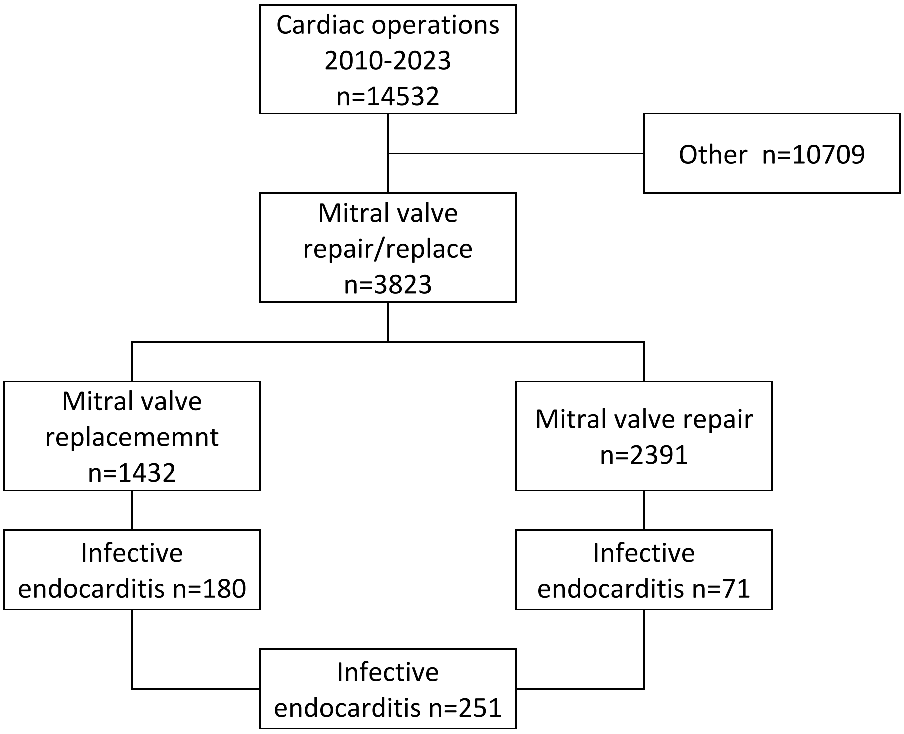

Figure S2. Receiver Operator Characteristic for time between diagnosis and surgery in relation to valve repair/replace

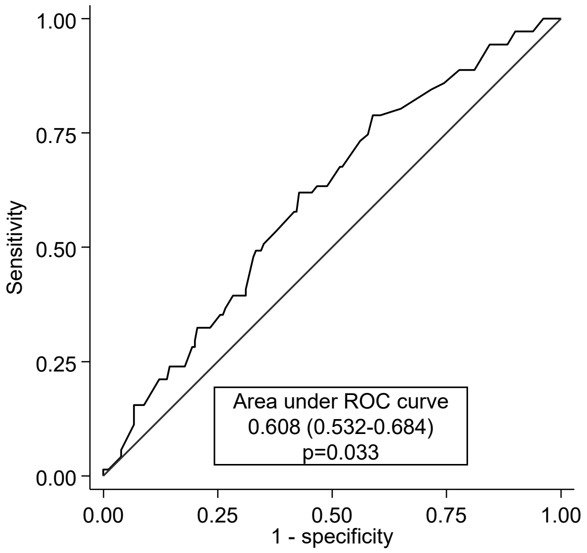

Table S1. Preoperative characteristics.

	MVR	MVr	p	MVR, weighted %/mean	MVr, weighted %/mean	ASMD post IPTW
N	180	71		120	67	120 VS 67
Hypertension, n (%)	118 (65.6)	36 (50.7)	0.032	60.1	57.4	0.055
Diabetes, n (%)	47 (26.1)	12 (16.9)	0.139	18.2	19.2	0.025
Obesity, n (%)	36 (20.0)	8 (11.3)	0.139	14.3	16.4	0.057
COPD, n (%)	27 (15.0)	5 (7.0)	0.097	10.0	11.2	0.038
Ejection fraction%, median (IQR)	60 (54-62)	57 (55-65)	0.625	57.8	58.2	0.037
Redo, n (%)	76 (42.2)	23 (32.4)	0.197	34.8	40.8	0.123
Peripheral arterial disease, n (%)	22 (12.2)	7 (9.9)	0.667	9.9	11.8	0.061
Previous Neurological Disease, n (%)	43 (23.9)	12 (16.9)	0.309	18.1	13.6	0.112
Unstable Angina, n (%)	0 (0.0)	1 (1.4)	0.283	0 (0.0)	0 (0.0)	/
Cardiogenic Shock, n (%)	14 (7.8)	3 (4.2)	0.410	4.6	6.6	0.086
Heart failure, n (%)	59 (32.8)	18 (25.4)	0.289	27.4	25.5	0.042
Pre-operative intubation, n (%)	19 (10.6)	3 (4.2)	0.139	5.1	6.8	0.066
PAPs>50, n (%)	22 (12.2)	7 (9.9)	0.667	9.2	8.9	0.008
Chronic Kidney Disease (Creat>2.0), n (%)	27 (15.0)	8 (11.3)	0.546	9.8	7.5	0.069
Dialysis, n (%)	8 (4.4)	4 (5.6)	0.745	4.5	2.8	0.077
Permanent pacemaker, n (%)	12 (6.7)	1 (1.4)	0.118	2.7	3.6	0.047
Multi valve, n (%)	73 (40.6)	40 (56.3)	0.025	50.6	43.7	0.139

COPD, Chronic Obstructive Pulmonary Disease; IQR, interquartile range; MVR, mitral valve prosthesis; MVr, mitral valve repair, PAPs, systolic pulmonary artery pressure.

Table S2. Postoperative outcomes.

	MVR	MVr	Coef.* (95%CI), p
Pneumothorax, n (%)	5 (2.8)	3 (4.2)	1.217 (0.251; 5.898), p=0.808
Embolism, n (%)	2 (1.1)	0 (0.0)	/
MOF, n (%)	11 (6.1)	4 (5.6)	0.735 (0.159; 3.398), p=0.694
Pacemaker implantation, n (%)	13 (7.2)	6 (8.5)	0.948 (0.253; 3.551), p=0.936
CCA, n (%)	2 (1.1)	1 (1.4)	1.053 (0.064; 17.405), p=0.971
AF, n (%)	57 (31.7)	20 (28.2)	1.321 (0.563; 3.100), p=0.522
Low cardiac output, n (%)	38 (21.1)	6 (8.5)	0.378 (0.084; 1.697), p=0.204
IABP, n (%)	7 (3.9)	3 (4.2)	0.873 (0.142; 5.352), p=0.883
ECMO, n (%)	1 (0.6)	0 (0.0)	/
Respiratory failure, n (%)	43 (23.9)	8 (11.3)	1.006 (0.312; 3.241), p=0.992
Stroke, n (%)	11 (6.1)	1 (1.4)	/
TIA, n (%)	4 (2.2)	1 (1.4)	0.270 (0.027; 2.703), p=0.265
AKI, n (%)	40 (22.2)	8 (11.3)	0.393 (0.130; 1.190), p=0.098
CVVH, n (%)	18 (10.0)	5 (7.0)	0.535 (0.146; 1.956), p=0.344
Major bleeding, n (%)	10 (5.6)	1 (1.4)	0.205 (0.024; 1.773), p=0.150
Reoperation for bleeding, n (%)	17 (9.4)	3 (4.2)	0.935 (0.215; 4.056), p=0.928
Wound Deheiscence, n (%)	2 (1.1)	1 (1.4)	4.780 (0.416; 54.876), p=0.209

AF, Atrial Fibrillation; AKI, Acute kidney injury; AVR, aortic valve replacement, CABG, coronary artery bypass grafting; CCA, cardiocirculatory arrest; CPB, cardiopulmonary bypass; CVVH, Continuous venovenous hemofiltration; ECMO, extracorporeal membrane oxygenation; IABP, intra-aortic balloon pump; IQR, interquartile range; LVEF, left ventricular ejection fraction; MOF, Multiorgan Failure; MVR, mitral valve prosthesis; MVr, mitral valve repair; TIA, transient ischemic attack.
